# The Poly(acrylonitrule-*co*-acrylic acid)-graft-β-cyclodextrin Hydrogel for Thorium(IV) Adsorption

**DOI:** 10.3390/polym9060201

**Published:** 2017-05-31

**Authors:** Guojian Duan, Qiangqiang Zhong, Lei Bi, Liu Yang, Tonghuan Liu, Xiaoning Shi, Wangsuo Wu

**Affiliations:** 1 College of Pharmacy, Gansu University of Chinese Medicine, Lanzhou 730000, China; duangj2004@126.com (G.D.); shixn2017@163.com (X.S.); 2 Radiochemistry Laboratory, School of Nuclear Science and Technology, Lanzhou University, Lanzhou 730000, China; zhongqq201701@163.com (Q.Z.); bil16@lzu.edu.cn (L.B.); yangl15@lzu.edu.cn (L.Y.); wuws@lzu.edu.cn (W.W.)

**Keywords:** β-cyclodextrin, functional hydrogel, adsorbent material, Th(IV)

## Abstract

In this report, the β-CD(AN-*co*-AA) hydrogel was used to remove the thorium(IV) [Th(IV)] from the water system, and the new adsorbent was characterized through Fourier transform infrared spectroscopy (FTIR), scanning electron microscopy (SEM), and X-ray diffraction (XRD). The influences of contact time, pH value, ionic strength, solid-liquid ratio, initial Th(IV) concentration, and temperature on Th(IV) adsorption onto the functional hydrogel were researched. The results showed that the experimental data followed the Langmuir isotherm and the maximum adsorption capacity (*q*_max_) for Th(IV) was 692 mg/g at pH 2.95, which approached the calculated (*q*_e_) 682 mg/g. The desorption capacity of Th(IV) in different HNO_3_ concentrations ranging from 0.005 to 0.5 M was also studied, and the percentage of the maximum desorption was 86.85% in the condition of 0.09 M HNO_3_. The selectivity of β-CD(AN-*co*-AA) hydrogel was also be studied, the results indicated that this material retained the good adsorption capacity to Th(IV) even when the Ca^2+^, Mg^2+^, or Pb^2+^ existed in the system. The findings indicate that β-CD(AN-*co*-AA) can be used as a new candidate for the enrichment and separation of Th(IV), or its analogue actinides, from large-volume solution in practical application.

## 1. Introduction

With economic development the problem of energy shortages becomes more and more serious and nuclear power has been used significantly in production and living. Thorium, as a potential nuclear fuel, has attracted much attention [1]. The use nuclear power generates spent nuclear fuel, which contains various fission products, one of them is thorium. Generally, the direct toxicity of thorium is low because of its stable character at room temperature [2], but if the concentration of Th(IV) ions in the solution is large enough, it will arouse acute toxicological effects for people, which may lead to potential occupational carcinogens and progressive or irreversible renal injury [3]. At present, there are also some important tetravalent actinides, such as Np(IV), U(IV), and Pu(IV), which appear in the use of nuclear fuel processes, but those are instable in tetravalent form, so the stable thorium can be studied as an model element for others [4,5,6,7].

A series of technologies have been introduced to remove thorium from aqueous solutions, such as chemical precipitation, ion exchange, evaporation, and adsorption [8]. Among these methods, adsorption is one of the most widely applied in radiation/nuclear chemistry [2,9,10]. In recent years, many groups have synthesized new materials to separate Th(IV) from the solution [11,12,13].

In order to expand the scope of the adsorption materials, the adsorption behavior of Th(IV) ions onto β-Cyclodextrin (β-CD) derivatives is studied. β-CD is a cyclic oligosaccharide connected by α-(1,4) glycoside bonds which consist of seven glucose units. Meanwhile, the secondary hydroxyl groups and primary hydroxyl groups form the outer side and the narrow side, respectively, which forms the β-CD as a truncated cone. This conformation gives the β-CD hydrophobic internal cavity and hydrophilic external surface at the same time, which can easily form inclusion complexes with types of guest molecules through non-covalent interactions and, therefore, provides ideal binding sites [14,15,16,17,18]. These new complexes have excellent solubility, bioactivity, and stability because of the special structure with the guest molecules, which leads to serious applications in food, cosmetics, and even in pharmaceutical industries. However, very few studies on the adsorption behavior of metal ions onto the functionalization of β-CD [19] have been conducted. Meanwhile, the adsorption mechanism is still unclear.

The aim of the present research was to study the efficiency of β-CD(AN-*co*-AA) for removing thorium from wastewater. Fourier transform infrared spectroscopy (FTIR), scanning electron microscopy (SEM), and X-ray diffraction (XRD) were used to characterize the structure of β-CD(AN-*co*-AA). The effect of various experimental parameters, including the pH of the solution, contact time, initial thorium concentration, and temperature, as well as adsorption kinetics, isotherm models, and thermodynamics, were studied. In addition, the properties of reusability and selectivity of β-CD(AN-*co*-AA) were also investigated.

## 2. Materials and Methods

### 2.1. Materials

All chemicals used in our experiment were purchased as analytically pure and no further purification was conducted.

### 2.2. Synthesis of Poly(acrylonitrule-co-acrylic acid)-graft-*β*-cyclodextrin Hydrogel (*β*-CD(AN-co-AA))

This hydrogel was synthesized via the formation of C-C single bonds between the AN and AA, ester bonds between the hydroxyl groups (O-H) in β-CD, and the carboxyl groups (COOH) in the AA. Briefly, β-CD (0.7495 g, 0.7 mmol) was added to acetic acid (100 mL) and stirred for 10 min under 60 °C. NaHSO_3_ (0.1040 g, 1.0 mmol) was added to the above solution and stirred for 15 min under the protection of nitrogen. Then, AN (2.6712 g, 50.4 mmol) and AA (3.6318 g, 50.4 mmol) were added to the above reaction dropwise at the same time. The solution was stirred for 10 min, and K_2_S_2_O_8_ (0.3244 g, 1.2 mmol) was added to the reaction system one time. The reaction was stirred for 3.0 h at 60 °C under the protection of nitrogen. Then, the reaction system was cooled to room temperature and let stand for 0.5 h. Pouring the supernatant out, the white precipitate was washed with distilled water and ethanol three times, respectively. Then the solid product was dried in a vacuum oven at 40 °C for 24 h. In order to avoid the variations in characteristics dependent on the preparation, a 10 g lot of β-CD(AN-*co*-AA) hydrogel was prepared to conduct the overall investigations.

### 2.3. Characterization of *β*-CD(AN-co-AA) Hydrogel

#### 2.3.1. Fourier Transform Infrared Spectroscopy

**Figure 1:**
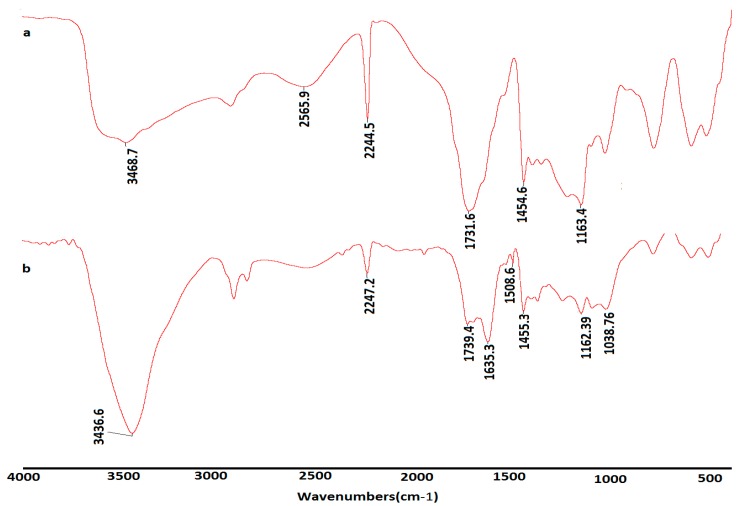
FTIR spectra of β-CD(AN-*co*-AA) (**a**) and β-CD(AN-*co*-AA)-Th^4+^ (**b**).

IR spectra of β-CD(AN-*co*-AA) and its Th(IV) complex were recorded on a FTIR spectrometer (Nicolet Avatar 360, Nicolet Instrument Corportion, Danbury, CT, USA). The powder of the sample was mixed with potassium bromide (KBr) and compressed into a transparent KBr tablet. The spectra were shown at wavenumbers from 4000 to 400 cm^−1^. The results of the FTIR analysis (Figure 1) showed the major adsorption bonds at the following frequencies. A broad and intense peak at 3469 cm^−1^ is attributed to O–H vibrations [20]; the –C≡N stretching vibrations peaks are appeared at 2244 cm^−1^; The peaks at 1732 and 1453 cm^−1^ are assigned to the C=O and C–O–C stretching bonds of the ester group [21,22].

In the spectrum of Th(IV) complex, the intense peak of 1732 cm^−1^ decreased, but the peak of 1455 cm^−1^ did not change obviously, which means a part of the acrylic acid groups were involved in the combination, while H–O–H bending vibrations of lattice water molecules in the complexes were expressed at 1635 cm^−1^.

#### 2.3.2. Scanning Electron Microscopy Measurements

To learn the morphology of the hydrogel composite particles, SEM measurements were carried on the gold-sputtered specimens with a JSM-6701F instrument (Jeol, Tokyo, Japan). Before the experiment, the sample was prepared by dripping a small amount of the particle suspension onto a cover glass and allowing it to air-dry.

**Figure 2:**
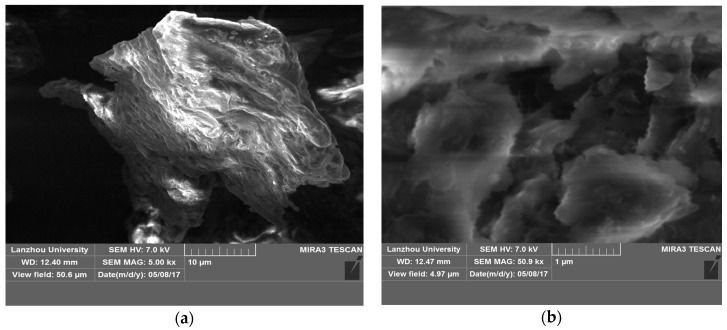
SEM of β-CD(AN-*co*-AA) hydrogel particles in different resolution size 10 μm (**a**) and 1 μm (**b**).

Figure 2 shows SEM images of the β-CD(AN-*co*-AA) hydrogel. The morphology of β-CD(AN-*co*-AA) resembled a cloud sheet, and the size of the gel sheet is 2 μm in length and 1 μm in width, mostly. SEM results indicated that the hydrogels’ structure provided a larger specific surface area, which is better for its combining with metal ions by providing wider exposed sites for adsorption, and the adsorption experiments also strongly demonstrated these results.

#### 2.3.3. X-ray Diffraction Measurements

**Figure 3:**
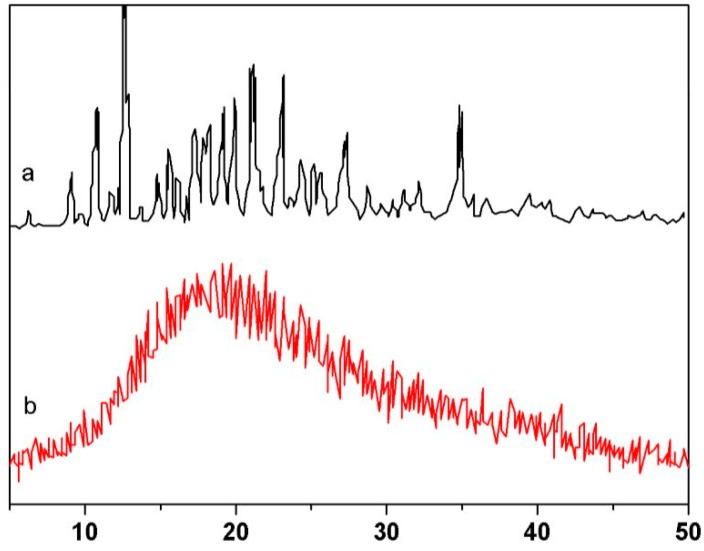
XRD pattern of β-CD (**a**) and β-CD(AN-*co*-AA) (**b**).

Powder X-ray diffraction is a method that can provide insightful information about the combination mode between the β-CD, AN, and AA. The XRD pattern of β-CD(AN-*co*-AA) was obtained using a powder X-ray diffractometer (Rigaku, The Woodlands, TX, USA) with copper K_α_ radiation at 150 mA and 40 kV, and the diffraction intensity data were scanned with a step size of 0.02° in the 2θ range from 5° to 50°. Figure 3 shows the XRD patterns of β-CD and β-CD(AN-*co*-AA). The XRD pattern of β-CD(AN-*co*-AA) showed a broad peak, which was consistent with its amorphous feature [23] and, at the same time, a number of intense and sharp peaks shown in the XRD pattern of β-CD [24] proved the crystalline characteristic of β-CD. This further indicates that these three materials, combined by chemical bonds, have formed a hydrogel, and β-CD has lost its crystalline characteristic.

### 2.4. Adsorption Experiments

The Th(IV) aqueous solution of known concentration was added to 10 mL polyethylene tubes, with a certain quantity of adsorbent, finally the mixed solution was 5.0 mL in batch experiments. HNO_3_ (0.1 M) or NaOH (0.1 M) was used to adjust the initial pH of the solution. The system was centrifuged at high speed (1.2 × 10^4^ r/min) for 20 min after vibrating at room temperature for the required time. A certain volume of supernatant was detected to determine the Th(IV) concentration by using a spectrophotometer (Perkin-Elmer, Waltham, MA, USA) at a wavelength of 662 nm, and the adsorption capacity of the β-CD(AN-*co*-AA) hydrogel of Th(IV) was calculated by the changes between the initial and final equilibrium concentrations.

### 2.5. Desorption Experiment

In order to examine the adsorption and desorption behaviors of thorium ions from β-CD(AN-*co*-AA) hydrogel, to ensure the reusability of adsorbents, at the end of adsorption experiments the suspension was centrifuged and all of the supernatant was poured out and then washed with distilled water. An equal volume of acid solution was added, and the suspension was shaken for 4 h. The amount of Th(IV) ions released into the solution was monitored using the same spectrophotometric method as in the adsorption experiments. The Th(IV) ions’ recovery percentage upon desorption from the hydrogel was calculated using the initially adsorbed Th(IV) ion amount and the amount found in the solution at the end of the desorption process, according to Equation. (1):

(1) $$\mathrm{Recovered}\;(\%)=\;\frac{q_{e}-q_{d}}{q_{e}}\times 100$$

where *q*_e_ is the number of adsorbed Th(IV) ion (g/g) and *q*_d_ is the amount of desorbed Th(IV) ion (g/g). The adsorption/desorption cycles were repeated ten times using the same batch of the hydrogel (10 mg).

## 3. Results and Discussion

### 3.1. Adsorption Performance Study

#### 3.1.1. Adsorption Kinetics

**Figure 4:**
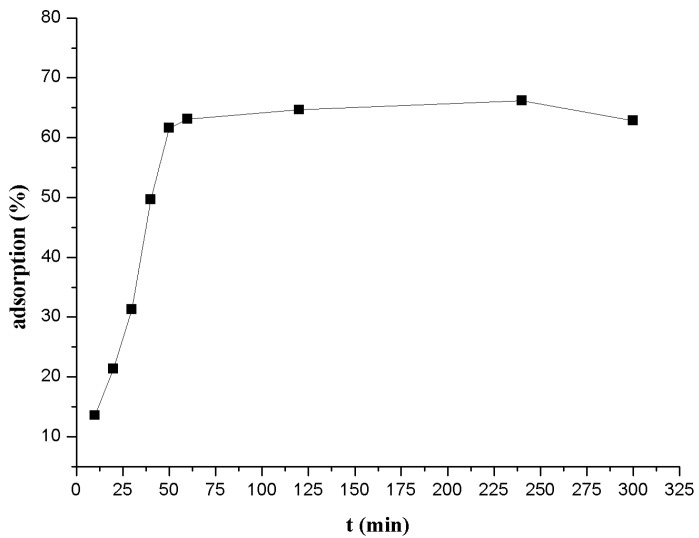
Effect of equilibrium time on Th(IV) adsorption onto β-CD(AN-*co*-AA).

Equilibration time is a key factor which has a direct relationship with the adsorption kinetics of adsorbent at the set initial adsorbate concentration. Figure 4 shows the impact of the contact time on Th(IV) adsorption onto β-CD(AN-*co*-AA), from the results of the experiment we can determine that the speed of Th(IV) adsorption onto β-CD(AN-*co*-AA) is very fast in the initial stage (about 30 min). The initial steep adsorption becomes slow, subsequently, attributed to the Th^4+^ ions (with a radius of about 0.102 nm), nearly an equilibrium state between the solution and the adsorbing material. These speed differences of Th(IV) adsorption onto β-CD(AN-*co*-AA) and desorption from the material are smaller and smaller as the time increases. This phenomenon will help us to understand the adsorption modality (chemical adsorption or physical adsorption) [25,26]. In order to ensure the adsorption equilibria were reached, the stir time was set for 4.0 h for all remaining batch experiments.

**Figure 5:**
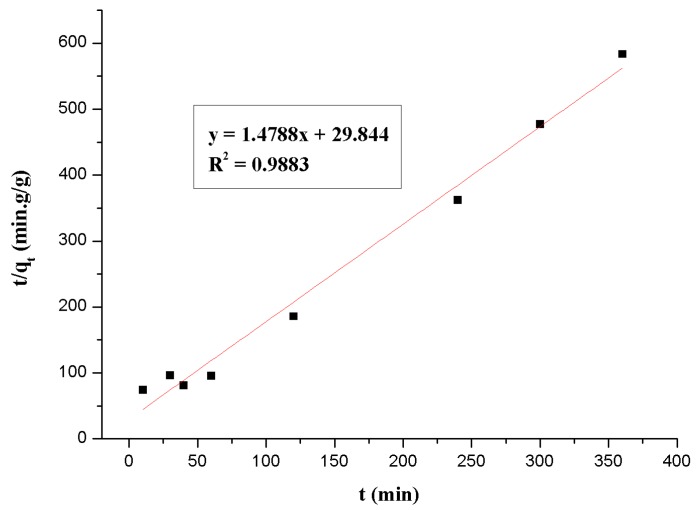
Test of pseudo-second-order adsorption kinetics plot for Th(IV).

In order to describe the kinetic mechanism clearly, which was controlled by the process of Th(IV) adsorption onto β-CD(AN-*co*-AA), the pseudo-second-order models was used to test the experimental data. This model is given as:

(2) $$\frac{t}{q_{t}}=\frac{1}{kq_{e}^{2}}+\frac{t}{q_{e}},$$

where *q*_t_ (g/g) is the amount of Th(IV) adsorbed on β-CD(AN-*co*-AA) at time *t*, and *q*_e_ (g/g) is the equilibrium adsorption capacity, *k* (g/(g∙min)) represents the second-order rate constant of the kinetic model. Figure 5 shows the results of using the Equation (2) to fit the experimental data. The equation of pseudo-second-order is *y* = 1.4788*x* + 29.844; as is known from the calculation, according to the slope and intercept, *k* is 0.0660 g/(g∙min); the *q*_e_ equal to 0.6820 g/g and the linear correlation coefficient *R*^2^ = 0.9883. These results show that the pseudo-second-order rate equation is a suitable method to describe the kinetic adsorption.

#### 3.1.2. Effect of pH on Th(IV) Adsorption onto β-CD(AN-*co*-AA)

**Figure 6:**
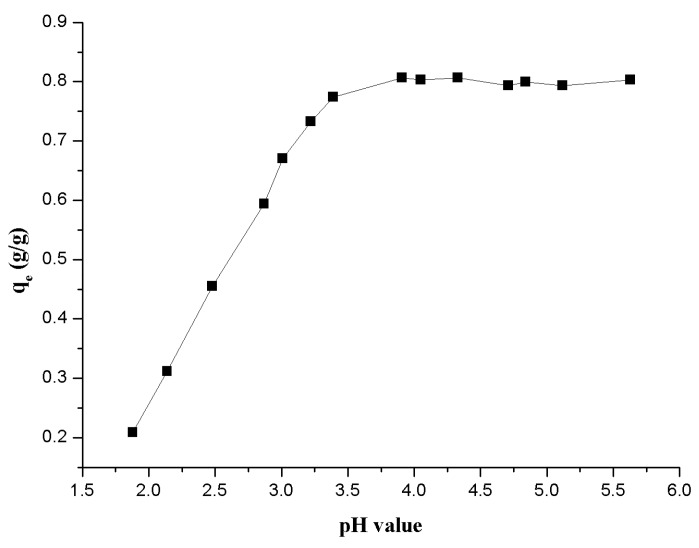
Effect of the pH value of the solution on the adsorption of Th(IV) onto β-CD(AN-*co*-AA).

The pH value of the adsorption system is an important parameter which is a direct impact on the uptake of metal ions from aqueous solution. At the same time, it affects the adsorbent surface charge, the ionization degree and the adsorbate speciation [27,28]. From Figure 6, the pH value of the aqueous solution directly affects the adsorption results of Th(IV) onto β-CD(AN-*co*-AA). The adsorption progress of Th(IV) onto β-CD(AN-*co*-AA) can be divided into two stages. The first is the adsorption percentage (from ~25% to ~95%) of Th(IV) onto β-CD(AN-*co*-AA), increasing with the changing of pH value (from 1.8 to 4.0) of adsorption system. The second is the adsorption percentage in a stable range (from ~90 to ~95%) with the changing of pH value (from 4.0 to 5.6) of adsorption system. The changed trend of stage one may be attributed to the surface properties of β-CD(AN-*co*-AA), such as the surface charge and the ionization of surface functional groups of β-CD(AN-*co*-AA). The best result of adsorption in the pH range of 3–4 may be due to the formation of the Th(IV) complexes with carboxyl groups of the acrylic acid unit and hydroxyl groups of the β-CD unit on the surface of β-CD(AN-*co*-AA). It is well known that, increasing with the pH, the more carboxylic groups tend to deprotonate, and the more carboxylate ions form stable complexes with Th(IV) ions. At the same time, this operation of decreasing the positive charge density of the adsorption edges increases the adsorption capacity of this kind of hydrogel through electrostatic attraction [29]. There are certain form features of the Th(IV) complex in a specific solution pH which may be important factors that affect the uptake efficiency of β-CD(AN-*co*-AA) for Th(IV) adsorption [30]. According to the hydrolysis constants of Th(IV) in aqueous solution, in the range of 2.0 to 4.0 of the pH value, Th^4+^ and [Th(OH)]^3+^ are the main species [7,31]. If the value of the solution pH is over 4.0, Th(OH)_4_ is gradually becoming the main species. Therefore, the good result of Th(IV) onto β-CD(AN-*co*-AA) may be attributed to the solid Th(OH)_4_ on the surface of the hydrogel. We take into account both of the factors of adsorption percent and Th(IV) species present at different pH values and, in the following experiments, pH = 2.95 ± 0.05 was selected as the value of the experiment systems.

#### 3.1.3. Effect of Ionic Strength

**Figure 7:**
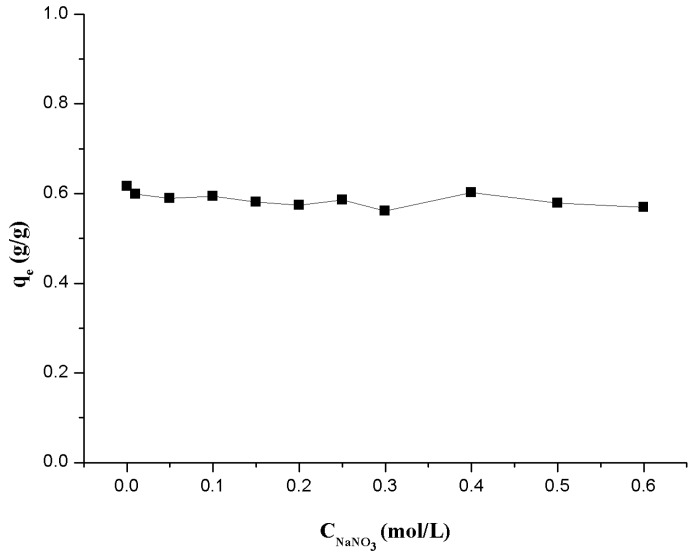
Effect of the ionic strength of the solution on the adsorption of Th(IV) onto β-CD(AN-*co*-AA).

Figure 7 shows the relationship between ionic strength and the adsorption ability of Th(IV) onto β-CD(AN-*co*-AA) hydrogel. From the results we can find that the adsorption results of Th(IV) onto β-CD(AN-*co*-AA) do not change significantly when the ionic strength increases from 0.00 to 0.60 mol/L NaNO_3_ at a pH value in the range of 2.95 ± 0.05, which indicates that, under this experimental condition, the inner-sphere surface complexes between Th(IV) and β-CD(AN-*co*-AA) has been formed [32,33]. Therein, ion exchange has no contribution to the adsorption of Th(IV) onto β-CD(AN-*co*-AA). Combined with the previous contents, the adsorption depends on the pH value and is independent of the ionic strength.

#### 3.1.4. Effect of Solid-Liquid Ratio

**Figure 8:**
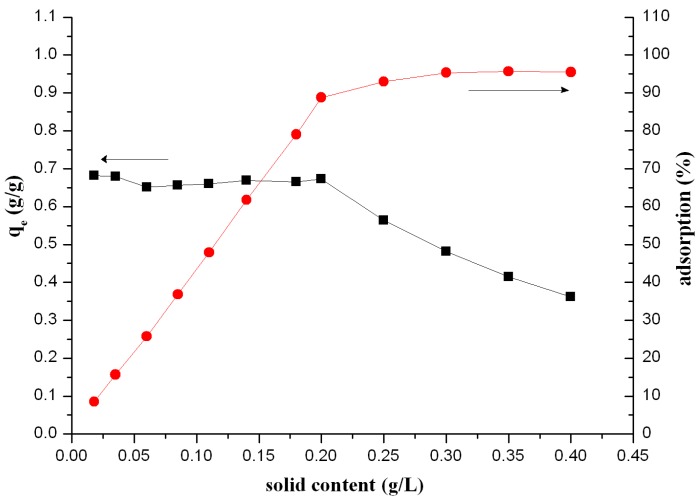
Effect of solid content on the adsorption of Th(IV) onto β-CD(AN-*co*-AA), square point show the value of *q*_e_ (g/g) and dot show the value of “adsorption %”.

The relationship between adsorbent content and adsorption percentage of Th(IV) from the aqueous solution onto β-CD(AN-*co*-AA) is shown in Figure 8. From the results it is known that the adsorption ratio of Th(IV) onto β-CD(AN-*co*-AA) is kept at a relatively stable value with the solid content increasing in the range of 0.008~0.2 g/L. Then the adsorption ratio decreases with the solid content increasing when the solid content exceeds 0.2 g/L. However, the concentration of Th(IV) adsorbed onto β-CD(AN-*co*-AA) gives the results that firstly increase, and then become stable with the increasing hydrogel content (the turning point is at 0.2 g/L, as well). These results can be due to two reasons [34]. First, the Th(IV) can be easily combined with the sites of the hydrogel and, thus, we obtain a high absorption rate. This is because the adsorption of the per unit mass of β-CD(AN-*co*-AA) is kept at a high level when the solid content is relatively low, ranging from 0.008–0.2 g/L in this experimental condition, and all of the adsorption sites are saturated when the solid-liquid ratio is below 0.2 g/L, so the *q*_e_ is similar with each other and the adsorption percentage has significantly increased. Secondly, the adsorption percentage can approach the maximum with enough adsorbent. However, in this aspect the content of Th(IV) is fixed, and when the β-CD(AN-*co*-AA) is over 0.2 g/L, there will not be enough metal ions to combine with the adsorbent, so we can find the *q*_e_ is decreased with the increasing amount of β-CD(AN-*co*-AA), while the percent of absorption is at its maximum.

#### 3.1.5. Effect of Initial Th(IV) Concentration

**Figure 9:**
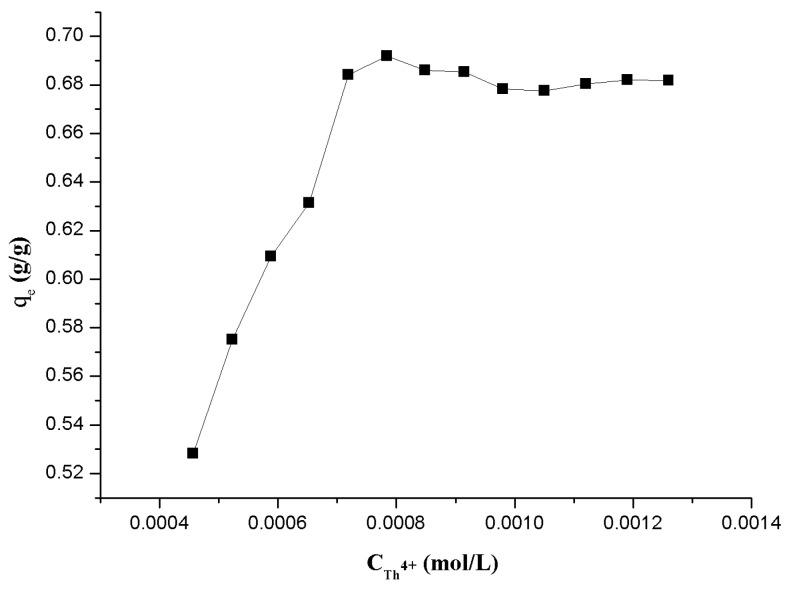
Effect of the initial concentration of Th^4+^ on the adsorption behavior onto β-CD(AN-*co*-AA).

The initial concentration of Th(IV) directly affects the adsorption process and efficiency. To study the relationship between the adsorption and the metal ions concentration in the aqueous phase will help us to understand the adsorption behavior. Therefore, the initial concentration of Th(IV) varied within the range of 3.92 × 10^−4^~1.26 × 10^−3^ mol/L, with the other parameters being kept constant. Figure 9 shows the trend of adsorption capacity (g/g) with the increase of the initial metal concentration. The changing curve indicates that, in the range of 3.92 × 10^−4^ to 7.19 × 10^−4^ mol/L of the Th(IV) concentration, the adsorption amount increases with the metal concentration. Combined with the above results of Section 3.1.4, the larger part of the active sites was used in the adsorption process when more Th(IV) ions were contained in the reaction system. However, if the Th(IV) concentration exceeded 7.19 × 10^−4^ mol/L, adsorption capacity will saturate the available binding sites on the adsorbent. Under the present experimental conditions, the maximum adsorption reached was 0.692 g/g.

#### 3.1.6. Adsorption Isotherm

The adsorption isotherms are mathematical models when the adsorption process reaches an equilibrium state which is used to describe the distribution of the adsorbate species between the liquid and adsorbent. In this report, the Langmuir and Freundlich models are the most commonly used to describe the adsorption equilibrium isotherms. If there are some important facts, such as the adsorption is localized in a monolayer and the adsorbate molecules do not interact with each other, the adsorption isotherm uses the Langmuir model, whereas the Freundlich isotherm model is always used as an empirical relationship which assumes that there are many adsorption energies in different adsorption sites to describe the adsorption of solutes from a liquid to a solid surface.

The Langmuir isotherm equation was used in the linearized form [27]:

(3) $$C_{e}/q_{e}=1/(q_{\max}\cdot b)+C_{e}/q_{\max},$$

where *b* and *q*_max_ are Langmuir constants related to adsorption energy and adsorption maximum, separately. A linearized plot of *C*_e_/*q*_e_ against *C*_e_ gives *b* and *q*_max_.

The Freundlich equation, based on the adsorption on a heterogeneous surface, is represented as follows [35]:

(4) $$\mathrm{lg}q_{e}=\mathrm{lg}K_{F}+(1/n)\mathrm{lg}C_{e},$$

where *K*_F_ and n are Freundlich the adsorption isotherm constants. *K*_F_ and *n* can be gained from the linear plot of lg*q*_e_ against lg*C*_e_.

**Figure 10:**
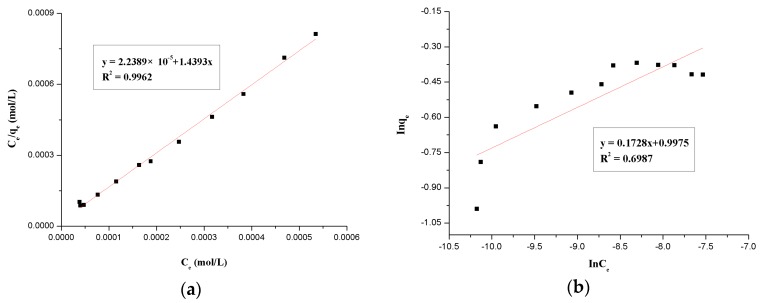
Adsorption isotherms of Langmuir model (**a**) and Freundlich model (**b**).

The linearized Langmuir and Freundlich plots are shown in Figure 10a,b, respectively. From these results, the Langmuir model is presented with higher correlation coefficients than the Freundlich model, so we can say that the Langmuir model is much better suited to describe the adsorption manner than the Freundlich model in the range of relevant concentration, combined with the results above, which means that the adsorption process matches with the chemisorption characteristics in a monolayer.

The essential feature of the Langmuir isotherm is that it can be described as a dimensionless constant separation coefficient, *R*_L_, which is used to express whether the adsorption system is “favorable” or “unfavorable”. The separate coefficient, *R*_L_ is given as [36]:

(5) $$R_{L}=1/(1+bC_{0}),$$

where *C*_0_ and *b* are the initial Th(IV) concentration (mg/L) and Langmuir adsorption constant (L/mg), respectively. Table 1 shows the results of the computation. Based on the impact of the separation factor on the form of the isotherm, the *R*_L_ values are significantly smaller than one, which indicates that the β-CD(AN-*co*-AA) hydrogels are a favorable adsorbent to Th(IV) [37].

#### 3.1.7. Effect of Temperature

**Figure 11:**
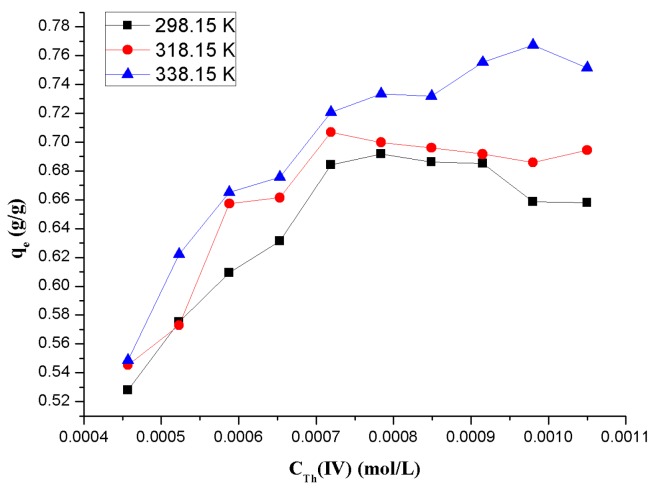
Effect of temperature on the adsorption of Th(IV) onto β-CD(AN-*co*-AA).

In order to further study the adsorption pattern of Th(IV) ions on β-CD(AN-*co*-AA), a series of experiments were present at different temperatures, such as 298.15, 318.15, and 338.15 K, which was used to indicate the effect of temperature, with the concentration of Th(IV) ranging from 4.45 × 10^−4^ to 1.05 × 10^−3^ mol/L, and all other parameters constant at their optimum values. From Figure 11 we can know that the temperature rise has improved the adsorption abilities, which represents that the adsorption is an endothermic process. In order to ensure the thermodynamic features, three basic thermodynamic parameters, enthalpy change (Δ*H*^0^), entropy change (∆*S*^0^), and free energy change (∆*G*^0^), were calculated according to Equations (6) [38] and (7):

∆*G*^0^ = −RTln*K*^0^ = −*RT*ln(55.5*K*) (6)

∆*G*^0^ = ∆*H*^0^ − *T*∆*S*^0^ (7)

where *K*^0^ (dimensionless) is the standard equilibrium constant calculating the standard free energy changes (∆*G*^0^) of the adsorption processes, and is equal to 55.5 K; *R* and *T* are the gas constant (*R* = 8.314 J/(mol·K)) and the temperature, respectively. The values of ∆*H*^0^ and ∆*S*^0^ were calculated from the slopes and intercepts of the linear variation of ∆*G*^0^ versus T. By the results shown in Table 2, we can find the ∆*H*^0^ < 0, which confirms that the process of the adsorption between β-CD(AN-*co*-AA) and Th(IV) ions was endothermic and the randomness in the solid-solution interface increased along with the adsorption process. Meanwhile, the values of ∆*G*^0^ < 0 are negative at all temperatures in these experiments, confirming that the adsorption process is spontaneous and thermodynamically favorable. The increasing adsorption by increasing temperature is another indicator that the adsorption process is a chemisorption process and is an endothermic process.

**Figure 12:**
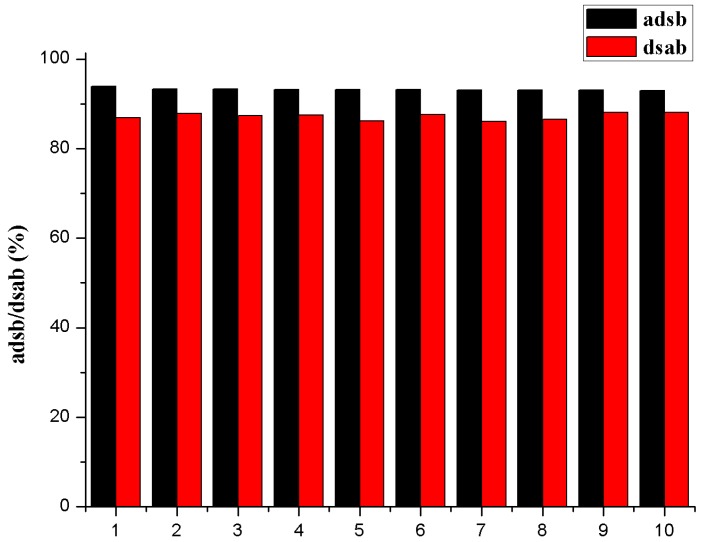
The amount of Th^4+^ adsorbed as a function of the adsorption-desorption cycle. Adsorption (adsb) and desorption (dsab) cycles: adsorption-sample volume = 5.0 mL, Th(IV) concentration = 6.5334 × 10^−4^ mol/L, adsorption dose (β-CD(AN-*co*-AA)) = 0.30 g/L, initial pH = 2.95 ± 0.05, equilibration time = 4 h; desorption-sample volume = 5.0 mL of 0.09 M HNO_3_.

Repeated availability of the bound metal ions is an important factor for an advanced adsorbent in the sorption process. In order to reduce the overall costs, those adsorbents should have outstanding adsorption and desorption properties at the same time. Considering the effect of pH on Th(IV) adsorption onto β-CD(AN-*co*-AA), and the acid hydrolysis reaction of the hydrogels, the study for the proper concentration of the acid solution selected from a series of HNO_3_, ranging from 0.005 to 0.5 M, were tested. The experimental results showed that a maximum 87.00% desorption could be achieved with 0.09 M HNO_3_ (Figure 12). In the first cycle, β-CD(AN-*co*-AA) hydrogels could adsorb 93.87% Th(IV), which could be desorbed up to 86.85%; in the second desorption cycle, the hydrogels which were desorbed from first cycle could adsorb 92.08% Th(IV) and desorbed up to 87.87%. Even in the tenth cycle, the percentage of adsorption and desorption could remain at 92.39% and 88.04%, respectively.

In this study, the strong acid proves a higher metal ion recovery, which indicates that the electrostatic attraction and chelated attraction are the most important aspects to control the adsorption of Th(IV) onto β-CD(AN-*co*-AA) hydrogel [39]. In addition, these results do not obviously improve the loss of the hydrogel’s adsorption capacity in ten adsorption/desorption cycles. The characteristic that the material can be reused several times makes the new adsorbent favorable in practical applications.

### 3.2. Adsorption Selectivity

**Figure 13:**
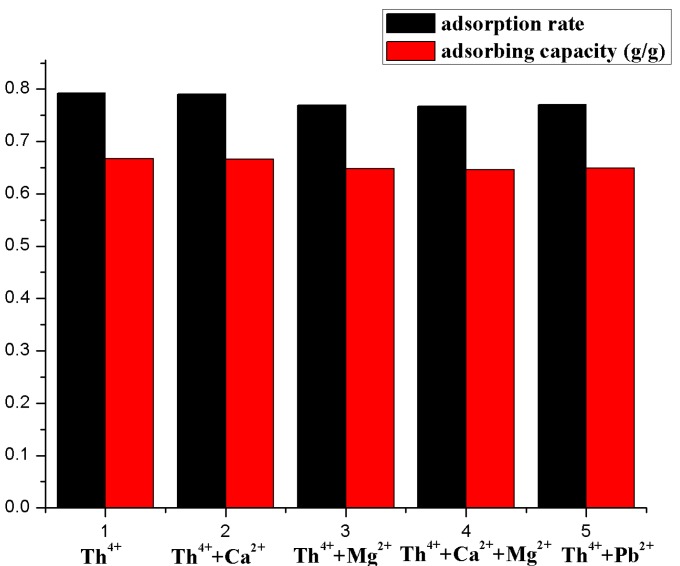
T Effect of interfering ions on the adsorption of Th(IV) onto β-CD(AN-*co*-AA).

As we know, there are various kinds of ions in industrial wastewaters and seawater, which might have a great effect on the adsorption process. Therefore, one of the important characters for an adsorbent is the selectivity. To better understand the selectivity of β-CD(AN-*co*-AA) towards Th^4+^, Ca^2+^, Mg^2+^, and Pb^2+^ were chosen as the interfering ions, and all of the competing ions often coexist with Th(IV) ions, showing certain interference suppressions in co-existing solutions. In this experiment, we fixed all of the initial concentrations at 4.03 × 10^−4^ mol/L, and we put 0.5 mL Th^4+^ solution into the centrifuge tube, then choose one or two of the three interfering ions, adding 0.5 mL into the centrifuge tube in order. Then we obtained the results and Figure 13 shows that when the solution consisted of interfering ions, the absorbability of the hydrogel to the Th^4+^ did not have a significant reduction, and the adsorption percentage remained in the range of 79.04~77.08%. This phenomenon indicated that β-CD(AN-*co*-AA) exhibited good adsorption selectivity for Th^4+^ rather than the other elements mentioned above, and the hydrogel can be probably used in the separation of Th^4+^ from industrial wastewater systems.

## 4. Conclusions

In this research β-CD(AN-*co*-AA), as an excellent absorbent separating the thorium ions from aqueous solution, was studied by a series of adsorption experiments. The separation of thorium relied on contact time, pH, and solid-liquid ratio. The optimum separation conditions were as follows: equilibration time of 4 h, pH value of 2.95 ± 0.05, solid-liquid ratio equal to 0.20 g/L, and a temperature of 298.15 °C. The adsorption process follows a pseudo-second-order model, and the adsorption equilibrium conforms to the Langmuir model. The adsorption type could be classified as chemical adsorption and surface complexation. Further systematic investigation is needed to fully understand the sorption mechanism. From the results of reusability and selectivity, this adsorbent material performs a perfect application in the field of chemical factors, especially for the nuclear science and technology to pre-concentrate or separate actinides from industrial wastewater.

## Figures and Tables

**Table 1:** *R*_L_ values based on the Langmuir isotherm.

No.	Initial Th^4+^	*R*_L_ Value
	Concentration (mmol/L)	
1	0.000327	0.0455
2	0.000392	0.0382
3	0.000457	0.0329
4	0.000523	0.0289
5	0.000588	0.0258
6	0.000653	0.0233
7	0.000719	0.0212
8	0.000784	0.0195
9	0.000849	0.018
10	0.000915	0.0167
11	0.00098	0.0156
12	0.00105	0.0147

**Table 2:** Thermodynamic parameters.

Temperature (K)	∆*H*^0^ (J/mol)	∆*S*^0^ (J/mol K)	∆*G*^0^ (kJ/mol)
298.15	−44.85	33.25	−9.96
318.15	−44.85	33.25	−10.62
338.15	−44.85	33.25	−11.29
