# Correction: Duan, G.J., et al. The Poly(acrylonitrule-*co*-acrylic acid)-graft-*β*-cyclodextrin Hydrogel for Thorium(IV) Adsorption. *Polymers* 2017, *9*, 201

**DOI:** 10.3390/polym12010227

**Published:** 2020-01-16

**Authors:** Guojian Duan, Qiangqiang Zhong, Lei Bi, Liu Yang, Tonghuan Liu, Xiaoning Shi, Wangsuo Wu

**Affiliations:** 1 College of Pharmacy, Gansu University of Chinese Medicine, Lanzhou 730000, China; duangj2004@126.com (G.D.); shixn2017@163.com (X.S.); 2 Radiochemistry Laboratory, School of Nuclear Science and Technology, Lanzhou University, Lanzhou 730000, China; zhongqq201701@163.com (Q.Z.); bil16@lzu.edu.cn (L.B.); yangl15@lzu.edu.cn (L.Y.); wuws@lzu.edu.cn (W.W.)

The authors wish to make the following corrections to their paper [1]: We have recently been made aware of some errors and omissions in the following paragraph of our recent paper.

1. The title of the original publication [1] is, “The Poly(acrylonitrule-*co*-acrylic acid)-graft-*β*-cyclodextrin Hydrogel for Thorium(IV) Adsorption”.

According to suggestions of reviewers, this has been modified as follows, “*β*-Cyclodextrin modified Poly(Acrylonitrile-*co*-Acrylic Acid) Hydrogel for Thorium(IV) Adsorption”

2. In the Section “2.3.1. Fourier Transform Infrared Spectroscopy” of the Materials and Methods:

On page 2, there is one mistake in the first sentence in the last paragraph. The information of FTIR spectrometer “Nicolet Avatar 360, Nicolet Instrument Corportion, Danbury, CT, USA” should be corrected to, “Nicolet Avatar 360, Nicolet Instrument Corportion, Connecticut, USA”.

On page 3, in the second paragraph, we have added more discussion and results. This paragraph should be corrected to, “In the spectrum of Th(IV) complex, the intense peak of 1732 cm−1 decreased, but the peak of 1455 cm−1 showed no obvious change, which means a part of the acrylic acid groups were involved in the combination. At the same time, the bond of O–H vibrations was changed from 3469 cm−1 to 3436 cm−1, which means that the Th(IV) chelated with the OH groups in β-CD of the copolymer [23]. The H–O–H bending vibrations of lattice water molecules in the complexes were expressed at 1635 cm^−1^.”

At the same time, we added a new Reference [23] to support our results in the second paragraph of page 3 and all the reference numbers after it have been changed accordingly. References [23–39] are now numbered [24–40].

3. In the Section “2.3.2. Scanning Electron Microscopy Measurements” of the Materials and Methods:

On page 3, there are some mistakes in the second sentence in the last paragraph of the original publication incorrectly state, “The morphology of *β*-CD(AN-*co*-AA) resembled a cloud sheet, and the size of the gel sheet is 2 μm in length and 1 μm in width, mostly”. Instead, this statement should read, “The morphology of *β*-CD(AN-*co*-AA) has a loose flocculent structure”.

4. In the Section “2.3.3. X-ray Diffraction Measurements” of the Materials and Methods:

On page 4, the third line in the first paragraph, we have modified the powder X-ray diffractometer’s type information from “Rigaku, The Woodlands, TX, USA” to “Rigaku, Texas, USA”.

The last sentence of this paragraph in the original publication is, “This further indicates that these three materials, combined by chemical bonds, have formed a hydrogel, and *β*-CD has lost its crystalline characteristic.” To express the sentence more clearly, we would like to replace it with the following: “This further indicates that the product of copolymer is not a simply physical mixture.”

5. In the Section “2.4. Adsorption Experiments” of the Materials and Methods:

On page 4, we have added some detailed information about our experimentation. The last sentence of this paragraph should be corrected to, “Concentration of Th(IV) was analyzed with the Arsenazo-III spectro photometric method on a spectrophotometer (Perkin-Elmer, Waltham, MA, USA). A quantity of 1 mL Th(IV)solution sample,1 mL 0.5 M HCl and 1.0 mL 0.1 % Arsenazo-III aqueous solution were added to a 25 mL glass flask, respectively, and the final solution volume was filled up to 25 mL by adding deionized water. After 15 min, the absorbance of the mixture liquid was measured at 662 nm, and the adsorption capacity of the *β*-CD(AN-*co*-AA) hydrogel of Th(IV) was calculated by the changes between the initial and final equilibrium concentrations.”

6. In order that readers understand our experiment more easily, we added detailed information about our experimental condition to some figures (from Figure 4 to Figure 9).

On page 5, the new title and conditions of Figure 4 should be, “**Figure 4.** Effect of equilibrium time on Th(IV) adsorption onto *β*-CD(AN-*co*-AA). ([Th(VI)]_0_ = 6.5334 × 10^−4^ M, pH = 2.95 ± 0.05, T = 298.15 ± 1.00 K, I = 0.50 M NaNO_3_).”

On page 6, the new title and conditions of Figure 5 should be, “**Figure 5.** Test of pseudo-second-order adsorption kinetics plot for Th(IV). ([Th(VI)]_0_ = 6.5334 × 10^−4^ M, pH = 2.95 ± 0.05, T = 298.15 ± 1.00 K, I = 0.50 M NaNO_3_).”

On page 7, the new title and conditions of Figure 6 should be, “**Figure 6.** Effect of the pH value of the solution on the adsorption of Th(IV) onto *β*-CD(AN-*co*-AA). ([Th(VI)]_0_ = 6.5334 × 10^−4^ M, m/V = 0.18 g/L, T = 298.15 ± 1.00 K, I = 0.50 M NaNO_3_).”

On page 7, the new title and conditions of Figure 7 should be, “**Figure 7.** Effect of the ionic strength of the solution on the adsorption of Th(IV) onto *β*-CD(AN-*co*-AA). ([Th(VI)]_0_ = 6.5334 × 10^−4^ M, m/V = 0.18 g/L, pH = 2.95 ± 0.05, T = 298.15 ± 1.00 K).”

On page 8, the new title and conditions of Figure 8 should be, “**Figure 8.** Effect of solid content on the adsorption of Th(IV) onto *β*-CD(AN-*co*-AA), squares show the value of q_e_ (g/g) and dots show the value of “adsorption %” ([Th(VI)]_0_ = 6.5334 × 10^−4^ M, pH = 2.95 ± 0.05, T = 298.15 ± 1.00 K, I = 0.50 M NaNO_3_).”

On page 8, the new title and conditions of Figure 9 should be, “**Figure 9.** Effect of the initial concentration of Th^4+^ on the adsorption behavior onto *β*-CD(AN-*co*-AA). ([pH = 2.95 ± 0.05, m/V = 0.18 g/L, T = 298.15 ± 1.00 K, I = 0.50 M NaNO_3_).”

These changes have no material impact on the conclusions of our paper. The authors would like to apologize for any inconvenience caused to the readers by these changes. 
